# The Prognostic Value of Cardiac Biomarkers in Patients with Acute Myocardial Infarction during and after Hospitalization

**DOI:** 10.31083/j.rcm2309320

**Published:** 2022-09-16

**Authors:** Karolina Idzikowska, Michal Kacprzak, Marzenna Zielinska

**Affiliations:** ^1^Department of Interventional Cardiology, Medical University of Lodz, 92-213 Lodz, Poland

**Keywords:** galectin-3, MR-proANP, cardiovascular disease, myocardial infarction, cardiac death, reinfarction

## Abstract

**Background:**

Myocardial infarction (MI) carries a strong risk of death and the development of major adverse cardiovascular events (MACE). A number of biomarkers have been proposed for risk stratification among patients with MI. The aim of this study was to determine whether elevated galectin-3 and midregional-pro atrial natriuretic peptide (MR-proANP) levels can be used as predictors of MACE in patients with acute myocardial infarction (AMI).

**Methods:**

Plasma levels of galectin-3 and MR-proANP were collected from 96 patients following their first AMI hospitalised in our clinic over the course of a year. Samples were taken on admission, and on the first and fifth day of hospitalization. During hospitalization, all patients were followed up for the occurrence of early major adverse cardiac events (MACE), defined as sudden cardiac arrest, new onset atrial fibrillation and need to use pressor amines. All patients were also followed up twelve months after AMI for the occurrence of late MACE defined as cardiac death, reinfarction and need for unscheduled PCI.

**Results:**

Patients who experienced early MACE had significantly higher galectin-3 and MR-proANP levels assessed on admission (*p* = 0.007, *p* = 0.003). ROC curve analysis found also galectin-3 concentration assessed on admission to be a strong predictor of late MACE (AUC = 0.75, *p* = 0.0061). MRproANP does not appear to have any value in predicting late MACE.

**Conclusions:**

A high concentration of galectin-3 and MR-proANP observed on admission in patients with acute myocardial infarction has significant prognostic value: it may identify patients at high risk of early adverse cardiac events after AMI. In contrast to MR-proANP, a high concentration of galectin-3 observed on admission may also identify patients at high risk of late MACE.

## 1. Introduction 

According to the fourth universal definition, myocardial infarction (MI) is an indication of myocardial necrosis in a clinical setting consistent with acute myocardial ischaemia [1]. Coronary artery disease (CAD) is the most common disease of the circulatory system in developed countries, with myocardial infarction and sudden cardiac death being the most common causes of death. MI affects men more often than woman, especially in the 40–55 year age group; however no such difference is apparent among seniors.

Myocardial infarction is typically diagnosed based on clinical, electrocardiographic and laboratory criteria. The typical biomarkers used in standard laboratory test panels include Troponin T (TnT) and Creatine Kinase Myocardial Band (CK-MB mass).

Despite the era of invasive treatment, MI carries a strong risk of major cardiovascular events and is often fatal. Therefore, in recent years, a number of emerging biomarkers have been proposed to facilitate risk stratification of patients with MI. These include myeloperoxydase (MPO) [2], human fatty acidbinding protein (h-FABP) [3], mid-regional pro-adrenomedullin (MR-proADM) [4], galectin-3 or midregional-pro atrial natriuretic peptide (MR-proANP).

Galectin-3 is a member of the lectin family. It is encoded by a single gene, *LGALS3*, located on chromosome 14, locus q21-q22 [5, 6]. The protein plays an important role in the regulation of many physiological and pathophysiological pathways, and is known to trigger inflammation in many conditions, such as diabetes and atherosclerosis [7]. In addition, galectin-3 expression is known to be associated with heart failure, inflammation, fibrogenesis and ventricular remodelling [8].

Pro-atrial natriuretic peptide (proANP) is a natriuretic peptide synthesized and secreted by cardiac muscle cells in the walls of the atria. It is known to play a role in natriuresis, diuresis and vasodilatation. It also counteracts the mechanisms that aggravate heart failure by stimulating prostaglandin synthesis, thus inhibiting the renin-angiotensin-aldosterone system, and reducing the secretion of antidiuretic hormone. MR-proANP has been found to demonstrate greater analytical stability and a longer half-life than ANP or its precursor fragments, and may hence be a more suitable marker [9, 10].

The aim of this study was to determine whether elevated galectin-3 and MR-proANP levels are predictors of MACE in patients with acute myocardial infarction (AMI).

## 2. Methods 

### 2.1 Study Population 

This prospective clinical study included 96 patients admitted to the Department of Interventional Cardiology of Medical University of Lodz, Poland. All had received a first diagnosis of acute myocardial infarction within one year. The inclusion criteria comprised STEMI or NSTEMI and invasive treatment of MI (primary PCI), while the exclusion criteria included age below 18 years or over 80 years, prior MI, the presence of chronic kidney disease (eGFR <30 mL/min/1.73 $m^{2}$) or history of cancer in the previous five years.

The study was approved by the local ethics committee (Medical University of Lodz). All patients gave their informed consent to participate in this study.

### 2.2 Study Protocol 

All patients qualified for the study underwent coronary angiography with PCI. All received standard pharmacological therapy used in myocardial infarction.

The following data was collected from all patients: sex, age and histories of hypertension, coronary artery disease, hypercholesterolemia (according to ESC guidelines), heart failure, diabetes mellitus (according to WHO diagnosis criteria), chronic kidney disease (eGFR 31–59 mL/min/1.73 $m^{2}$) and cigarette smoking. Blood pressure, heart rate and haemodynamic state were also collected. During hospitalization, all patients received echocardiography examination: ejection fraction was calculated using Simpson’s method. Standard laboratory tests for myocardial infarction were also carried out.

During hospitalization, all patients were followed up for the occurrence of early major adverse cardiac events (MACE), defined as sudden cardiac arrest, new onset atrial fibrillation and need to use pressor amines. Patients were also followed up twelve months after AMI for the occurrence of late MACE, defined as cardiac death, reinfarction and need for unscheduled PCI.

### 2.3 Galectin-3 and MR-proANP Assay 

Three sets of venous blood samples were collected during hospitalization: on admission to hospital, and on day one and day five of hospitalisation. The blood samples were centrifuged and then frozen and for storage at –20 °C until assay.

Galectin-3 assay was performed by sandwich ELISA, using a commercial kit by BioVendor (Laboratorni medicina a.s), with a sensitivity of 0.29 ng/mL. Although the assay detects both natural and recombinant Galectin-3; however no cross reactivity or interference was detected.

The MR-proANP level in the blood sample was determined by automated immunofluorescent assay (BRAHMS MR-proANP KRYPTOR) with a sensitivity of 10 pmol/L. The assay detects human MR-proANP; however, no significant cross-reactivity or interference was observed between human MR-proANP and analogues.

### 2.4 Statistical Analysis 

Categorical variables were summarized as frequencies with percentage values. The Shapiro-Wilk test was used to assess the normality of continuous variables. Any non-normal distributions were subjected to non-parametric tests and presented as medians with interquartile range.

Correlations were assessed using Spearman’s rank correlation coefficient. Continuous variables were compared using the Mann-Whitney U-test, and categorical variables with the chi-squared test with Yates’s correction for continuity. To assess the suitability of selected biomarkers in predicting MACE, receiver operator characteristic curves were constructed. Univariate logistic analysis and stepwise multiple regression were used to determine the predictive value of biomarkers in predicting MACE.

Statistical analysis was performed using STATISTICA 13.3 (StatSoft Inc, Tulsa, OK, USA). A *p*-value < 0.05 was assumed as statistically significant.

## 3. Results 

### 3.1 Characteristics of the Population 

The study included 96 patients, most of whom were men (67%). Eighty-six percent of patients were in Killip class I. STEMI of the inferior wall was diagnosed in 53% of patients. A diagnosis of NSTEMI was noted among 23% of patients. As stated in coronary angiography, the majority of patients were diagnosed with two-vessel disease. The participants were experiencing hyperlipidemia, hypertension or diabetes. Other study group features are presented in Table 1.

**Table 1.** **Patients characteristics, coronary angiography findings and concomitant treatment in whole study group on admission to hospital**.

Patients characteristics	N = 96
Age (years)	65 (58–71)
Men	64 (67%)
Heart rate	79 (65–90)
Systolic blood pressure [mmHg]	140 (124–160)
Type of MI	
STEMI inferior wall	23 (24%)
STEMI anterior wall	51 (53%)
NSTEMI	22 (23%)
Killip class	
I	83 (86%)
II–IV	13 (14%)
BMI	27 (24–30)
eGFR (mL/min/1.73 $m^{2}$)	84 (65–97)
Diabetes	22 (23%)
Heart Failure	9 (9%)
Hypertension	63 (66%)
Hyperlipidemia	35 (36%)
Family history of CAD	10 (10%)
Current or former smoker	53 (55%)
Results of coronarography	
One-vessel disease	35 (36%)
Two-vessel disease	36 (38%)
Three-vessel disease	25 (26%)
Concomitant therapy	
Aspirin	94 (98%)
Ticagrelor	43 (45%)
Clopidogrel	53 (55%)
GP IIb/IIIa blocker	60 (63%)
Statins	94 (98%)
Beta-blockers	86 (90%)
ARB	0 (0%)
ACEI	87 (91%)
Diuretics	34 (35%)

### 3.2 Levels of Galectin-3 and MR-proANP 

Mean galectin-3 plasma concentrations were higher on admission than on the first and fifth day of hospitalization: 12.56 ng/mL (9.9–20.7) on admission, 10.86 ng/mL (8.17–16.5) on day one of hospitalization, and 9.01 ng/mL (7.06–13.32) on day five.

Weak correlations were observed between heart rate on admission and the concentrations of galectin-3 on day one (*p* = 0.022, R = 0.23) and day five of hospitalization (*p* = 0.010; R = 0.26).

Neither sex, age, blood pressure, Killip class on admission, history of hypertension, hyperlipidemia, renal failure, lung disease, new-onset atrial fibrillation or obesity had any effect on galectin-3 levels, neither on admission, nor on day one or day five of hospitalization. However, patients with diabetes demonstrated significantly higher values of galectin-3 at all three time points (*p* = 0.020, *p* = 0.013, *p *< 0.001).

Patients with impaired systolic LV function had higher values of galectin-3 than patients with preserved systolic LV function (*p *< 0.001, *p* = 0.001, *p *< 0.001). Also, a weak relationship was observed between GFR assessed on admission and galectin-3 level, both assessed on admission (*p* = 0.01; R = 0.25) and on the fifth day of hospitalization (*p* = 0.04; R = –0.28). A stronger relationship was observed between galectin-3 and MR-proANP at all three time points (*p* = 0.002, R = 0.30 on admission; *p *< 0.001, R = 0.36 on day one; *p* = 0.002, R = 0.30 on day five). In addition, a relationship was observed between NT-proBNP level on day five and galectin-3 concentration at all three time points (*p* = 0.03, R = 0.30 on admission; *p *< 0.001, R = 0.36 on day one; *p *<0.001, R = 0.36 on day five).

MR-proANP plasma concentration was 151.2 pmol/L (99.9–282.7) on admission, 99.03 pmol/L (81.6–174.7) on day one of hospitalization and 104.6 pmol/L (78.6–164.8) on day five.

Neither heart failure, prior myocardial infarction, history of hypertension, diabetes, obesity, heart rate or sex had any effect on MR-proANP levels at any of the three measurement points.

Patients with normal systolic LV function had lower values of MR-proANP than patients with impaired systolic LV function (*p* = 0.005, *p *≤ 0.001, *p *≤ 0.001).

### 3.3 Early Major Adverse Cardiac Events 

During hospitalization, eighteen individuals developed early MACE. The univariate logistic regression analysis found that among the tested parameters (including heart rate, glucose and creatinine levels, NT-proBNP, MR-proANP, galectin-3, CRP among others), only galectin-3, MR-proANP, NT-proBNP and CRP assessed on admission turned out to be significant predictors of early MACE (*p* = 0.0103, *p* = 0.0028, *p* = 0.0254, *p* = 0.0112 respectively; Table 2).

**Table 2.** **Logistic regression analyses for predicting early MACE**.

Parameter	OR	95% CI	*p* value
*Univariable regression*			
CRP [mg/L]	1.016	1.004–1.028	0.0112
NT-proBNP on admission [pg/mL]	1.000	1.000–1.000	0.0254
Galectin-3 on admission [ng/mL]	1.044	1.010–1.078	0.0103
MR-proANP on admission [pmol/L]	1.005	1.002–1.008	0.0028
*Multivariable regression*			
CRP [mg/L]	1.018	1.004–1.031	0.011
Galectin-3 on admission [ng/mL]	1.054	1.015–1.095	0.007
MR-proANP on admission [pmol/L]	1.005	1.002–1.009	0.003

**Fig. 1.**
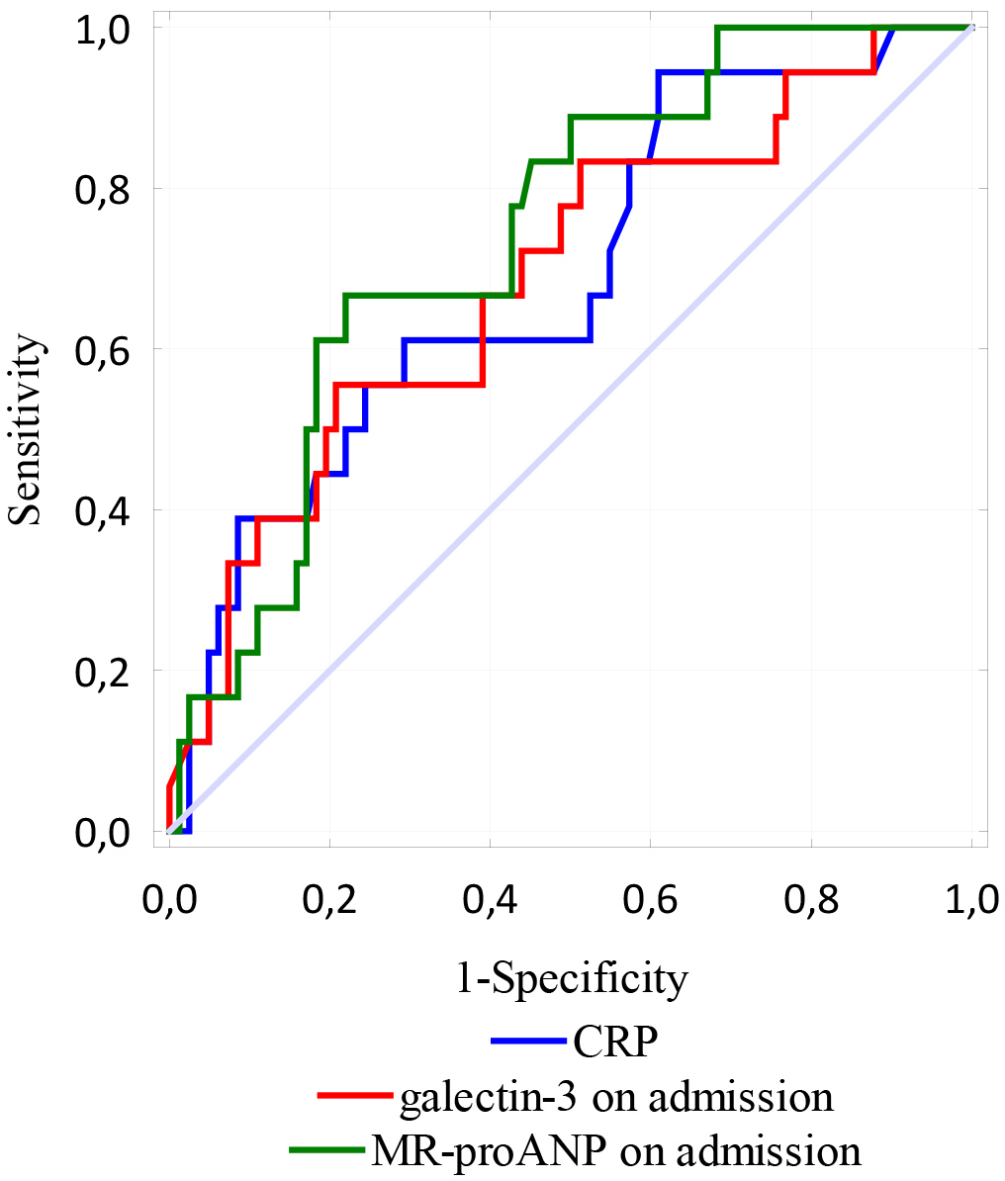
**ROC curves - variables tested: the concentrations of significant predictors of early MACE**.

ROC curve analysis found the concentration of CRP, galectin-3 and MR-proANP assessed on admission to be strong predictors of MACE during hospitalization (AUC = 0.688, *p* = 0.0064; AUC = 0.689, *p* = 0.0072; AUC = 0.742, *p* = 0.0000 respectively) (Fig. 1).

Stepwise forward logistic regression analysis found CRP, galectin-3 and MR-proANP assessed on admission to be significant predictors of early MACE (*p* = 0.011, *p* = 0.007, *p* = 0.003; Table 2).

### 3.4 Late Major Adverse Cardiac Events 

A follow-up conducted 12 months after the MI found nine individuals to have developed late MACE. Three patients had experienced reinfarction, four had undergone unscheduled coronary angioplasty and four had died.

**Fig. 2.**
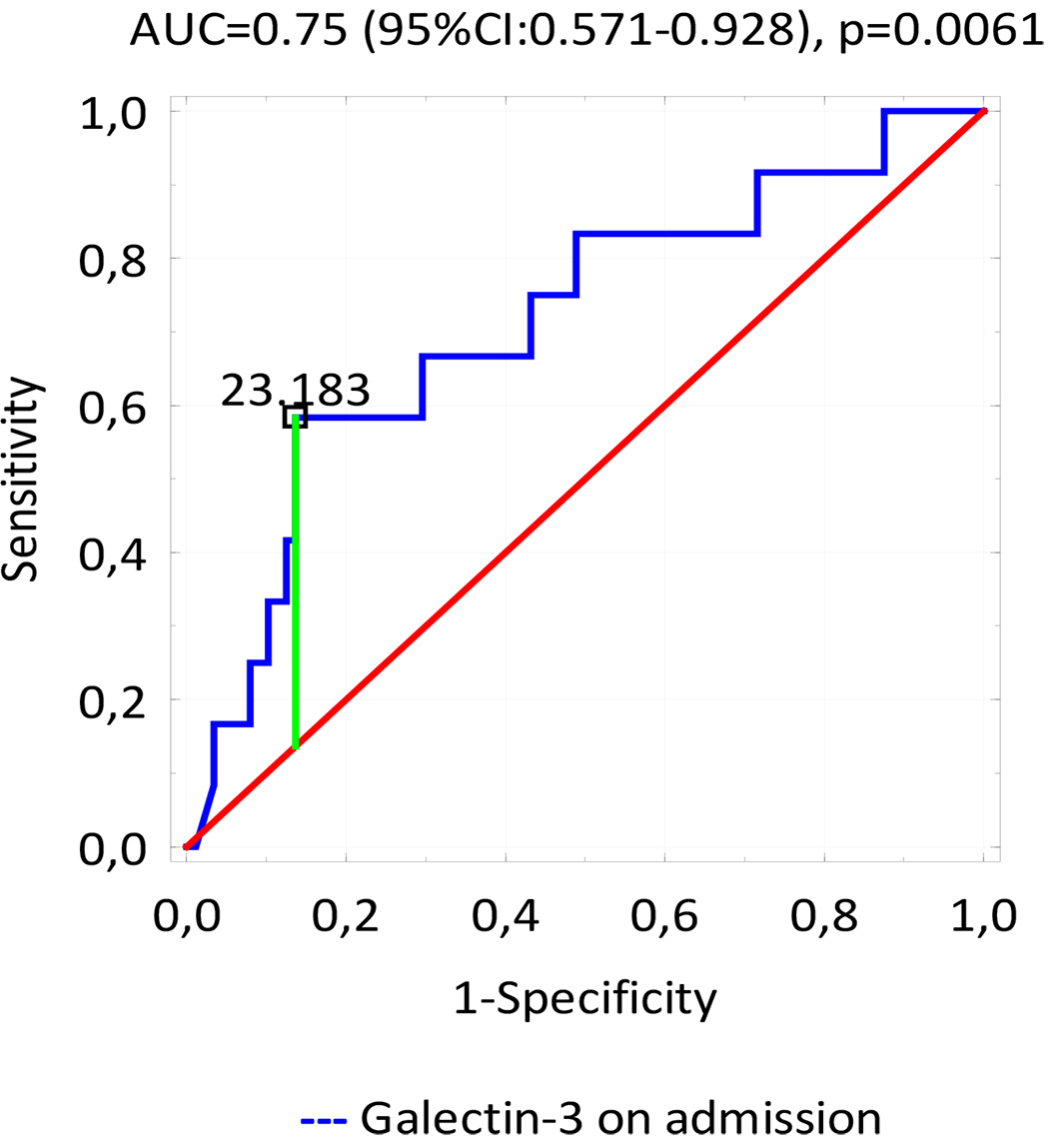
**ROC curve - variable tested: the concentrations of significant predictors of late MACE**.

No significant differences in the concentration of galectin-3 on day one of hospitalization were found between patients who experienced late MACE and uneventful survivors (*p* = 0.56). ROC curve analysis found galectin-3 concentration assessed on admission to be a strong predictor of MACE 12 months after discharge (AUC = 0.75, *p* = 0.0061) (Fig. 2); in this case, the galectin-3 cut-off value was 23.183 ng/mL (95% CI 2.664–54.059). In addition, ROC curve analysis for each major adverse cardiac event revealed that galectin-3 concentration collected on admission may be also used as a strong predictor of death (AUC = 0.854, *p *< 0.001).

In univariate logistic regression analysis, among the many tested factors (including age, sex, obesity, new-onset AF, diabetes, ejection fraction, NT-proBNP and MR-proANP), only galectin3 collected on admission was found to be significant predictor of late MACE (*p* = 0.0297; Table 3); this was confirmed by stepwise forward logistic regression analysis (*p* = 0.0297; Table 3).

**Table 3.** **Logistic regression analyses for predicting late MACE**.

Parameter	OR	95% CI	*p* value
*Univariable regression*			
Galectin 3 on admission	1.044	1.004–1.085	0.0297
*Multivariable regression*			
Galectin 3 on admision	1.044	1.044–1.085	0.0297

No significant differences in MR-proANP concentration at any time point were observed between patients who developed late MACE and those who did not (*p* = 0.83, *p* = 0.76, *p* = 0.7). MRproANP was not found to be a significant factor predicting late MACE, either by univariate or stepwise forward logistic regression analysis.

Early and late MACE were also analysed together. Stepwise forward logistic regression analysis found CRP, galectin-3 assessed on admission and NT-proBNP assessed on day five to be significant predictors of both early and late MACE (*p* = 0.034, *p* = 0.007, *p* = 0.043; Table 4).

**Table 4.** **Logistic regression analyses for predicting early and late MACE**.

Parameter	OR	95% CI	*p* value
*Multivariable regression*			
CRP [mg/L]	1.014	1.001–1.027	0.034
Galectin-3 on admission [ng/mL]	1.047	1.012–1.083	0.007
NT-proBNP on the fifth day [pg/mL]	1.000	1.000–1.000	0.043

## 4. Discussion 

Our present study yielded two key findings. Firstly, high concentrations of galectin-3 and MR-proANP assessed on admission may be significant factors predicting early MACE in patients with AMI. In addition, in contrast to MR-proANP, a high concentration of galectin-3 on admission may also be a predictor of late MACE.

It has been suggested that MR-proANP could be used to predict cardiovascular death [11], and that it might be a predictor of all-cause mortality and MACE in patients with symptomatic CAD [12]. High plasma concentrations of MR-proANP have been found to effectively identify patients with higher risk of death/AMI at 360 days after episode of chest pain [13, 14]. However, in the present study, no relationship was found between MR-proANP level and the occurrence of MACE 12 months after AMI.

Numerous studies indicate a relationship between MR-proANP and chronic heart failure (HF). Indeed, increased levels of MR-proANP have been found to be associated with an increased risk of death in patients with HF [15, 16]. In addition, others confirm a relationship between MR-proANP level and acute HF [17, 18]. Patients with dyspnea also demonstrated higher levels of MR-proANP, and the addition of this biomarker to the standard laboratory test panel improved the diagnostic accuracy for acute HF. Furthermore, MR-proANP may be an indicator of impaired left ventricular function [19].

Our present findings confirm that patients with normal systolic LV function had lower values of MR-proANP than those with impaired systolic LV function. In addition, no relationship was found between galectin-3 level on day one of hospitalization and the occurrence of both early and late MACE after acute myocardial infarction. However, the presence of high plasma concentrations of galectin-3 measured on admission was a significant predictor of death after AMI.

Tsai *et al*. [20] found the concentration of galectin-3 assessed six hours after PCI to be a strong predictor of 30-day mortality among patients with STEMI undergoing primary PCI (*p *< 0.001). In the present study, to identify the peak level with greater accuracy, the concentration of galectin-3 was measured at three time points: i.e., on admission, and on days one and five of hospitalization. O’Donoghue *et al*. [11] also found increased levels of galectin-3 to be associated with an increased risk of cardiovascular death in patients with STEMI (*p *< 0.001); however, in contrast to our data, this relationship did not remain significant after adjusting for traditional risk factors.

Similarly to our present findings, Lisowska *et al*. [21] found galectin-3 to be a predictor of cardiovascular death after AMI in a group of 233 patients. In this case, the concentration of galectin-3 was assessed within 24 hours of admission. Patients with significantly higher mean concentrations of galectin-3 were more likely to die during the follow-up period (mean 2.8 years) (20.0 ng/mL vs 8.0 ng/mL; *p* = 0.0005). However, many more exclusion criteria were used than the present study, such as severe congestive heart failure and unstable haemodynamic state. Another study based on 1013 patients found galectin-3 to be a strong predicting factor of cardiovascular death among patients with stable CAD and AMI who underwent coronary angiography (HR 1.87: 95% CI 1.04–3.33; *p* = 0.036) [22].

Several studies have found galectin-3 concentration to be an independent predictor of mortality after MI. Tymińska *et al*. [23] found galectin-3 level to predict cardiovascular death in patients with first-time STEMI (*p* = 0.01); however, unlike our present study, galectin-3 concentration was measured only after 72 to 96 hours after hospital admission. Galectin-3 has been found to peak 12 hours after acute inflammatory stimulation [24]; this has been confirmed in a previous study, but with a mean follow-up of 5.4 years [25]. Our findings indicate that the concentration of galectin-3 assessed on admission was also a predictor of reinfarction; this is in line with Szadkowska *et al*. [26], who also report that galectin-3 may be a predictor of reinfarction early after first MI.

Our present findings suggest that patients with normal systolic LV function had lower values of galectin-3 than those with impaired systolic LV function. A previous study of 1342 patients with myocardial infarction found those with higher galectin-3 levels to have a higher risk of developing heart failure after MI [25]. High galectin-3 levels have also been found to be positively correlated with advanced congestive heart failure, but negatively correlated with LVEF (R= –0.253; *p *< 0.001) [20]. In a recent study, Węgiel *et al*. [4] report that galectin-3 level might not be a good predictor of adverse left ventricule remodelling. Over-expression of galectin-3 has been also associated with decompensated congestive heart failure [26], an increased risk of developing heart failure after myocardial infarction (OR = 2.1 95% CI 1.2–3.6; *p* = 0.010) [27] and worse prognosis in patients with EF >40% [28]. Two large trials (CORONA and COACH) based on patients with chronic and acute decompensated heart failure found that repeated measurements of galectin-3 levels provided significant prognostic value in identifying those with worse outcomes [29]. In contrast, Lisowska *et al*. [21] report that galectin-3 concentration did not correlate with EF value.

Our present findings confirm those of previous studies indicating that patients with diabetes tended to have higher levels of galectin-3 [30, 31, 32, 33]. Interestingly, galectin-3 levels have also been found to be associated with all-cause mortality and incident cardiovascular events in type 2 diabetes [34]. In addition, a relationship was observed between galectin-3 level and GFR in our group of patients; furthermore, higher levels of galectin-3 have previously been found to be associated with incident chronic kidney disease over a 10-year follow-up [35, 36] and with the progression of chronic kidney disease [37].

### Limitations of the Study 

Firstly, the present study is based on a relatively small group of patients with a relatively limited number of events. As such, further studies are needed to confirm our findings. Secondly, the study group was quite heterogeneous, including both STEMI and NSTEMI patients. Additionally, most patients were in Killip class I; however, we did not exclude any patients in a worse haemodynamic state. The study also excluded patients with previous MI or with conservative treatment of MI; as such, our findings do not apply to these groups.

##  5. Conclusions 

A high concentration of galectin-3 and MR-proANP observed on admission in patients with acute myocardial infarction has significant prognostic value: it may identify patients at high risk of early adverse cardiac events after AMI. In contrast to MR-proANP, a high concentration of galectin-3 observed on admission found also to be a significant factor predicting late MACE in patients with AMI. Although no single universal cardiac biomarker appears to exist for predicting MACE in patients with acute myocardial infarction, galectin-3 seems to be an effective predictor of both early and late MACE.

## Abbreviations

MI, myocardial infarction; AMI, acute myocardial infarction; STEMI, ST elevation myocardial infarction; NSTEMI, non-ST elevation myocardial infarction; MACE, major adverse cardiovascular events; ANP, atrial natriuretic peptide; MR-proANP, midregional pro-atrial natriuretic peptide; NT-proBNP, N-terminal prohormone of brain natriuretic peptide; MPO, myeloperoxydase; H-FABP, human fatty acid-binding protein; MR-proADM, mid-regional pro-adrenomedullin; CRP, C-reactive protein; GFR, glomerular filtration rate; PCI, percutaneus coronary intervention; LV, left ventricular; EF, ejection fraction; AF, atrial fibrillation; CAD, cardiovascular disease.
